# Impact of uORFs in mediating regulation of translation in stress conditions

**DOI:** 10.1186/s12860-021-00363-9

**Published:** 2021-05-16

**Authors:** Simone G. Moro, Cedric Hermans, Jorge Ruiz-Orera, M. Mar Albà

**Affiliations:** 1 Evolutionary Genomics Group, Research Programme on Biomedical Informatics, Hospital del Mar Medical Research Institute (IMIM) and Universitat Pompeu Fabra (UPF), Barcelona, Spain; 2 Bioinformatics Knowledge Center, Howest University of Applied Sciences, Bruges, Belgium; 3 Cardiovascular and Metabolic Sciences, Max Delbrück Center for Molecular Medicine in the Helmholtz Association (MDC), Berlin, Germany; 4 Catalan Institution for Research and Advanced Studies (ICREA), Barcelona, Spain

## Abstract

**Background:**

A large fraction of genes contains upstream ORFs (uORFs) in the 5′ untranslated region (5’UTR). The translation of uORFs can inhibit the translation of the main coding sequence, for example by causing premature dissociation of the two ribosomal units or ribosome stalling. However, it is currently unknown if most uORFs are inhibitory or if this activity is restricted to specific cases. Here we interrogate ribosome profiling data from three different stress experiments in yeast to gain novel insights into this question.

**Results:**

By comparing ribosome occupancies in different conditions and experiments we obtain strong evidence that, in comparison to primary coding sequences (CDS), which undergo translational arrest during stress, the translation of uORFs is mostly unaffected by changes in the environment. As a result, the relative abundance of uORF-encoded peptides increases during stress. In general, the changes in the translational efficiency of regions containing uORFs do not seem to affect downstream translation. The exception are uORFs found in a subset of genes that are significantly up-regulated at the level of translation during stress; these uORFs tend to be translated at lower levels in stress conditions than in optimal growth conditions, facilitating the translation of the CDS during stress. We find new examples of uORF-mediated regulation of translation, including the *Gcn4* functional homologue *fil1* and *ubi4* genes in *S. pombe.*

**Conclusion:**

We find evidence that the relative amount of uORF-encoded peptides increases during stress. The increased translation of uORFs is however uncoupled from the general CDS translational repression observed during stress. In a subset of genes that encode proteins that need to be rapidly synthesized upon stress uORFs act as translational switches.

**Supplementary Information:**

The online version contains supplementary material available at 10.1186/s12860-021-00363-9.

## Background

The analysis of ribosome profiling data has uncovered many translated small open reading frames (sORFs) that had hitherto remained hidden [4, 5, 7, 27, 44]. The set of translated sORFs includes sequences encoding proteins smaller than 100 amino acids in transcripts annotated as long non-coding RNAs [29, 45] as well as upstream ORFs (uORFs) located in mRNA 5′ untranslated regions (5’UTR) [28, 59]. Recent efforts have started to characterize the roles of sORFs at a genome scale [11, 23], but still much remains to be done.

Several uORFs have been found to repress the translation of the main coding sequence (CDS); this can happen for example by the dissociation of the two ribosomal subunits after uORF translation termination or by ribosome stalling at the uORF ([25]). As a large fraction of the mRNAs harbours uORFs in their 5’UTR, regulation of downstream translation by uORFs could potentially affect many genes and regulatory programs [10]. However, a global understanding of the impact of uORFs in translational regulatory is still lacking.

Previous studies have argued that uORFs are in general repressive because there is a negative relationship between the number of putatively translated uORFs in a transcript and the translational efficiency of the main coding sequence [12, 30]. These results are based on the comparison of different sets of genes, which can differ in other characteristics in addition to the number of uORFs. In order to better understand the effect of uORFs on CDS translation, studies that examine data from the same genes in different conditions are needed.

The number of ribosome profiling reads that map to an ORF can be used to estimate the level of translation of the sequence, as each read originates from one translating ribosome [8, 27]. Changes in the level of translation of a given CDS or uORF across two conditions can then be assessed by comparing the number of reads in each of the conditions, using the same approaches as when studying differential gene expression (DGE) with RNA-Seq data. These methods allow differentiating between genes that are primarily regulated at the level of transcription and those regulated at the level of translation [6, 26, 31]. The translational efficiency (TE) of a gene is defined as the ratio between the normalized number of mapped Ribo-Seq and RNA-Seq reads [28]. Changes in TE across conditions can also point to genes that are translationally regulated [56, 62].

Stress conditions result in major shifts in gene expression regulatory programs [19, 40]. In addition, the translation of most genes is severely impaired due the phosphorylation of the translation initiation factor eIF2, which causes a reduction in the amount of active eIF2-GTP-Met-tRNAi ternary complex for translation initiation [13, 49]. The translation of some specific genes, however, is activated during stress. On example is the transcription factor *Gcn4/ATF4*, a master regulator that activates the expression of several stress-response genes. The *S. cerevisiae Gcn4* mRNA contains four uORFs, the translation of the two uORFs that are closest to the CDS represses *Gcn4* protein synthesis but the first two uORFs are permissive for downstream translation [22, 24, 51]. After translation of the first two uORFs, the 40S remains attached to the mRNA, allowing ribosome scanning re-initiation [47]. In normal conditions this results in the translation of the two repressive uORFs but in stress conditions the association of the 40S with the ternary complex is delayed and the repressive uORFs are often bypassed, resulting in efficient translation of the downstream CDS.

Intriguingly, several genome-wide studies have reported that the ratio of Ribo-Seq reads in the 5’UTR with respect to the CDS is much higher in stress conditions than in normal conditions [16, 21, 27, 35]. This could mean that uORFs are translated more efficiently in stress conditions, resulting in repression of CDS translation. Alternatively, the pattern could also be generated by differences in the translation elongation rates of uORFs and CDS in the two conditions.

In order to better understand the effect of uORFs on regulating CDS translation, here we investigate the relative changes in uORF translation versus CDS translation in several stress experiments performed in *Saccharomyces cerevisiae* and *Schizosaccharomyces pombe.* We conclude that the translation of most uORFs is unlikely to affect the translation of the downstream coding sequence, at least in the conditions tested. The exception are mRNAs which are specifically up-regulated at the level of translation during stress, including a number of genes for which uORF-mediated regulation had not been previously reported.

## Results

### The translation of uORFs in relation to the CDS increases during stress

**Fig. 1:**
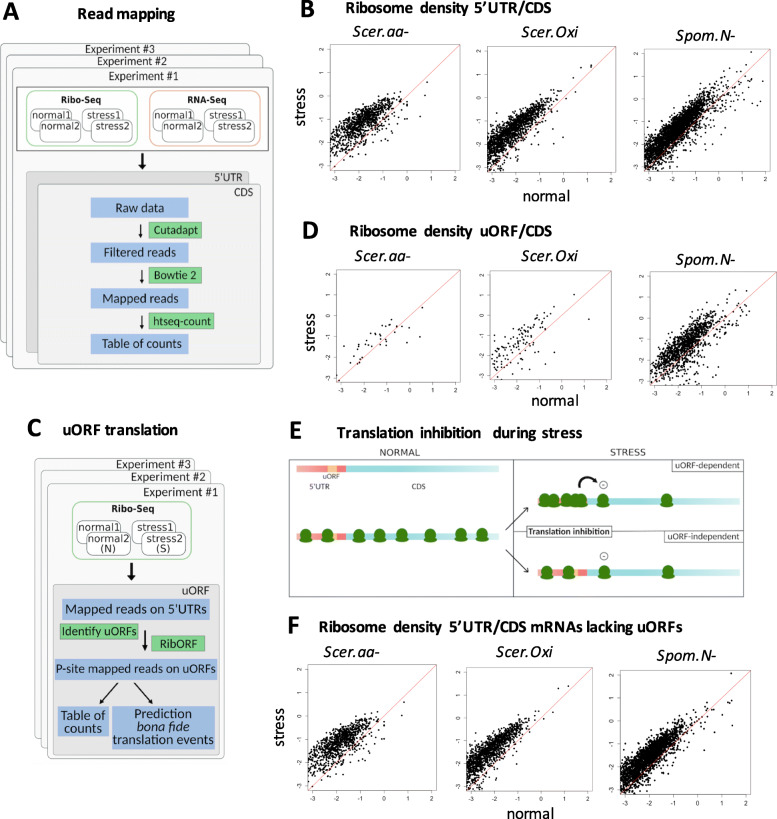
Increased ribosome density in 5’UTR versus CDS in stress conditions is independent of uORFs. **a** Workflow of 5’UTR and CDS read mapping and quantification. We analyzed Ribo-Seq and RNA-Seq data from three experiments: #1 *Scer.aa-*, amino acid starvation in *S. cerevisiae* [27], #2 *Scer. Oxi*, oxidative stress in *S. cerevisiae* [21] and #3 *Spom.N-*, nitrogen starvation in *S. pombe* [16]. For each experiment we used two normal replicates and two stress replicates. We mapped the sequencing reads to 5’UTR and CDS separately, obtaining the corresponding CDS and 5’UTR tables of counts for each gene and sample. **b** Log_10_ ratio of 5’UTR to CDS Ribo-Seq reads in stress versus normal conditions in the three experiments. Each data point represents a gene. The number of Ribo-Seq reads is a proxy of ribosome density. A relative increase in the density of ribosomes in the 5’UTR in stress can be observed for the majority of genes in the three experiments. We discarded genes with less than 10 average mapped reads in both conditions. Tables with CDS and 5’UTR reads were merged to calculate the ratios. **c** Workflow of uORF read mapping and quantification. We defined uORFs in the 5’UTRs as all ATG to STOP putative coding sequences of size 10 codons or longer. Subsequently we applied RibORF to identify the ribosomal P-site for each read, which corresponds to the tRNA binding site, and extracted the number of in-frame and out-of-mapped Ribo-Seq frame reads. The uORF Ribo-Seq table of counts was obtained by adding in-frame and out-of-frame reads for each uORF and sample; uORFs with less than 10 mapped reads, considering all samples together, were not considered for further analysis. **d** Log_10_ ratio of uORF to CDS Ribo-Seq reads in stress versus normal conditions in the three experiments. Each data point represents a gene. The number of Ribo-Seq reads is a proxy of ribosome density. Any uORFs with less than 10 Ribo-Seq mapped reads considering all samples together were discarded. Tables with CDS and uORF reads were merged to calculate the ratio. A relative increase in the density of ribosomes in uORFs in stress can be observed for the majority of genes in the three experiments. **e** proposed uORF dependent and uORF-independent mechanisms for the increase in relative ribosome density in the 5’UTR vs CDS in stress conditions. 1. uORF-dependent: uORFs are translated at higher levels during stress and this results in CDS translation repression. 2. uORF-independent: CDS translational arrest occurs independently of uORFs. **f** Same as B but for 5’UTRs not containing uORFs. No significant differences in the number of mRNAs with increased 5’UTR to CDS Ribo-Seq signal in stress were detected in subsets of 5’UTR containing or not containing uORFs with respect to the complete mRNA set, see Table S1 for additional information on the number of datapoints and proportions

We collected ribosome profiling (Ribo-Seq) and mRNA sequencing (RNA-Seq) from three different stress-inducing experiments, two performed in *Saccharomyces cerevisiae (*S*cer*) and a third one performed in *Schizosaccharomyces pombe* (*Spom*). In the first experiment, *Scer.aa-*, amino acids were depleted from the medium [27]; in the second one, *Scer. Oxi*, hydrogen peroxide (H_2_O_2_) was added to the medium [21]; and, in the third one, *Spom.N-*, starvation was induced by removing nitrogen from the medium [16]. For each experiment we obtained the raw RNA-Seq and Ribo-Seq sequencing reads and mapped them to the corresponding CDS and 5’UTR sequences, obtaining the relative number of mapped reads in each sequence (Fig. 1a, two replicates for each condition and experiment). Whereas the number of mapped RNA-Seq can be used to quantify mRNA levels, the number of mapped Ribo-Seq reads can be used as a proxy of ribosome abundance [27].

Next we compared the number of reads in the 5’UTR and the CDS in normal and stress conditions, taking the average between the replicates of the same condition. We observed a marked increase in the number of Ribo-Seq reads in the 5’UTR versus CDS in stress when compared to normal conditions, which was of 7.2 fold in *Scer.aa-,* 7.8 fold in *Scer. Oxi* and 2.8 fold in *Spom.N-,* when taking all genes together. This increase was consistent with that observed in previous reports [16, 21, 27]. When we plotted the number of reads in the 5’UTR and CDS for each gene it became clear that the observed increase in ribosome density in the 5’UTR during stress affected the majority of the genes (Fig. 1b, points above the diagonal). The RNA-Seq signal did not show such a bias (Figure S1), indicating that the results are related to changes in the translational status of the mRNAs.

We then identified putatively translated uORFs in the 5’UTRs using the RiboORF software (Fig. 1c) [29]. We focused on uORFs starting with AUG because all other possible initiation codons, including UUG or AUA, show a much lower translation initiation efficiency [14, 64]. To be considered, the uORFs had to be covered by at least 10 Ribo-Seq reads (taking all sequencing samples together). This resulted in 33 cases in *Scer.aa-*, 161 in *Scer. Oxi* and 1235 in *Spom.N-,* for which we had both uORF and CDS translation evidence. Differences between datasets were influenced by the size of the 5’UTRs, which are longer in *S. pombe* than in *S. cerevisiae*, and by the sequencing depth of the different experiments. We observed a similar increase in the number of of Ribo-Seq reads in uORFs versus CDS in stress conditions as that observed for the complete 5’UTR region (Fig. 1d). The excess of uORF translation in stress was of 4.2 fold in *Scer.aa-*, 5.8 fold in *Scer. Oxi* and 2.7 fold in *Spom.N-*. Similar results were obtained when we focused on uORFs with clear three nucleotide periodicity of the mapped Ribo-Seq reads and high homogeneity of the signal along the uORF (RibORF score > 0.7), which are clear signatures of translation (Figures S2 and S3). This data provided direct evidence of uORF translation, in contrast to previous studies on the possible role of uORFs in stress regulation, which did not analyze three nucleotide periodicity of the reads. Taken together, these results show that the level of translation of uORFs increases substantially during stress when compared to the CDS; in other words, they strongly suggest that the translation of uORFs is not inhibited to the same degree as the translation of the CDS.

As the translation of uORFs may be repressive for downstream translation, higher rates of translation of uORFs during stress could directly negatively impact CDS translation rates. This mechanism of CDS translation inhibition is illustrated in Fig. 1 (uORF-dependent). Alternatively, both 5’UTR scanning and uORF translation could be much less affected during stress than CDS translation, which could also lead to the observed bias (Fig. 1e, uORF-independent). Which of the two mechanisms may be the dominant one has not been investigated in earlier studies. To clarify the possible role of uORFs in the observed bias, we examined the data for the set of 5’UTRs lacking putatively translated uORFs. We found very similar results to those observed in the previous cases, both regarding the total number of reads (fold increase in 5’UTR in stress *Scer.aa-* 8.03, *Scer. Oxi* 7.4 and *Spom.N-* 4.2) as well as the per gene ratio distribution (Fig. 1f compared to Fig. 1b and d, see also Table S1 for more details), which favors the second scenario. In conclusion, although uORF-encoded peptides appear to be translated at higher rates during stress compared to the CDS, this is unlikely to have a major repressive effect on CDS translation.

### Changes in translational efficiency in the 5’UTR and the CDS are not inversely correlated

**Fig. 2:**
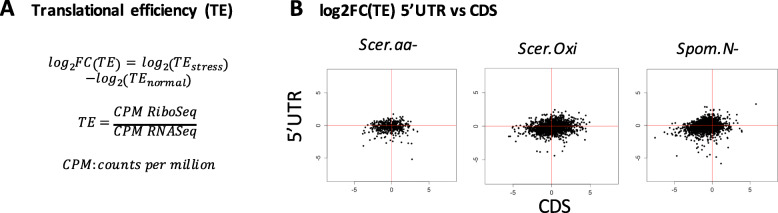
Changes in TE at the 5’UTR are not anti-correlated with changes in TE at the CDS. **a** Definition of changes in translational efficiency (TE). TE change is calculated as the log2TE between stress and normal conditions. **b** Correlation between log2TE in 5’UTR and CDS. We discarded genes with less than 10 mapped reads in both conditions, taking the average between replicates, in Ribo-Seq and/or RNA-Seq experiments Number of datapoints: *Scer.aa-* 438*; Scer. Oxi* 1503; *Spom.N-* 2039. Spearman correlation: *Scer.aa-* 0.046; *Scer. Oxi* 0.19; *Spom.N-* 0.27

Next we turned our attention to the relative changes in translational efficiency (TE) in stress versus normal conditions. TE was calculated as the ratio between the normalized number of mapped Ribo-Seq reads divided by the normalized number of mapped RNA-Seq reads, both in the 5’UTR and the CDS of each mRNA. The TE fold change (log_2_TE) represented the difference between TE in stress and TE in normal conditions (Fig. 2a). Contrary to what is to be expected if most uORFs were repressive, we found no anti-correlation in log_2_TE of the 5’UTR and the downstream CDS (Fig. 2b). Similarly, Gerashchenko et al. found that increased ribosome occupancy at the 5’UTR did not affect the TE of the downstream CDS [21]. We also observed that the spread of log_2_TE was higher in the CDS than in the 5’UTR, consistent with a smaller effect of stress on ribosome occupancy in the 5’UTR than in the CDS. The results for 5’UTR sequences containing putatively translated uORFs were very similar (Figure S4). These observations also support that the majority of uORFs do not play an important regulatory role in the translation of the CDS in stress.

### Translationally up-regulated genes show reduced uORF translation

Although the previous analyses suggest that most uORFs are unlike to regulate translation, several examples are known in which protein translation is modulated by uORFs during stress, such as the previously mentioned *Gcn4* master regulator gene [22, 24]. A functional term enrichment analysis indicated that uORFs are underrepresented among highly expressed genes and translation factors and over-represented among oxidative stress response genes (Table S2), pointing to specific roles in regulating this last set of genes.

**Fig. 3:**
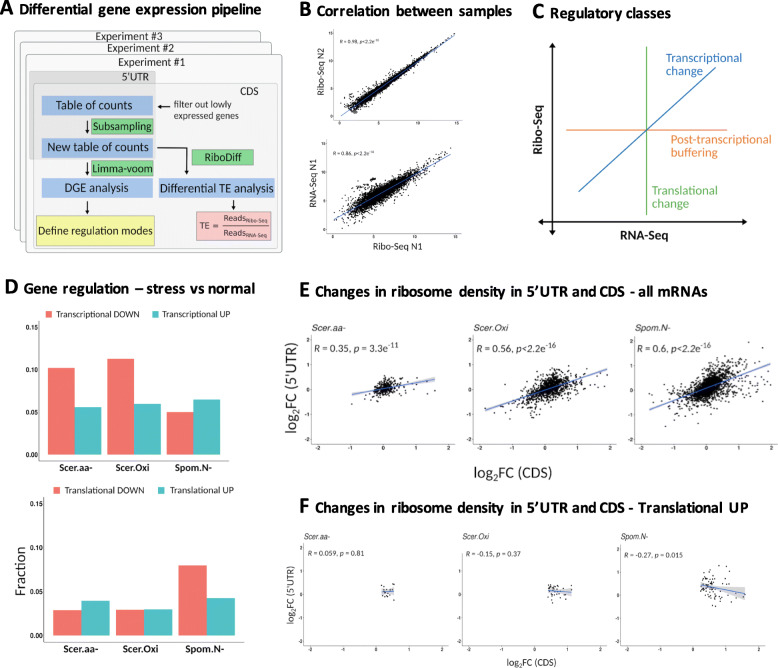
Identification of genes regulated at the transcriptional and translational levels during stress. **a** Workflow describing differential gene expression (DGE) and translational efficiency (TE) analyses using Ribo-Seq and RNA-Seq reads. In each experiment we subsampled the original table of counts as to have the same total number of reads in each Ribo-Seq and RNA-Seq sample considered. This ensured the results would not be biased by lack of statistical power in the samples with less coverage. The data was used to define regulatory classes for different sets of genes. **b** Correlation between replicates and between RNA-Seq and Ribo-Seq samples. Two representative examples are shown, data is counts per million (CPM). **c** Definition of regulatory classes after DGE analyses. Transcriptional change: Genes that showed significant up-regulation or down-regulation using both RNA-Seq and Ribo-Seq data. Translational change: Genes that showed significant up-regulation or down-regulation only with Ribo-Seq data. Post-transcriptional buffering: Genes that showed significant up-regulation or down-regulation only with RNA-Seq data. The axes represent logFC between stress and normal conditions. **d** Fraction of genes that showed translational or transcriptional changes. DGE was performed with the lima voom software and genes classified in the classes indicated in C. See Table S3 for more details on the number of genes and classes defined. **e** Significant positive correlation in ribosome density changes in the 5’UTR and the CDS for stress vs normal conditions. Data shown is for the complete set of mRNAs. log_2_FC (Fold Change) values based on the number of mapped Ribo-Seq reads, taking the average between replicates. **f** Same as E but for genes up-regulated at the level of translation. There is no positive correlation in this case

In order to better understand the possible roles of uORFs in translational regulation during stress, we performed differential gene expression (DGE) analysis of the mRNAs using the RNA-Seq and Ribo-Seq data separately (Fig. 3a). Gene expression levels were highly correlated between replicates of the same experiment and data type but the correlation decreased when we compared Ribo-Seq data against RNA-Seq data (Fig. 3b, Figure S5), as expected if there is some degree of translational regulation.

The combined DGE analysis defined three different sets of genes: 1. regulated at the level of transcription: genes that were significantly up-regulated or down-regulated in a consistent manner using both RNA-Seq and Ribo-Seq data; 2. regulated at the level of translation: genes that were only significant by Ribo-Seq and; 3. post-transcriptional buffering: genes that were only significant by RNA-Seq (Fig. 3c) [26]. We identified hundreds of genes in *S. pombe* and *S. cerevisiae* that were likely to be regulated at these different levels; transcriptional regulation encompassed 10–15% of the genes, and translational regulation 6–12% of the genes, depending on the experiment (Fig. 3d, Table S3). We found that ribosomal proteins and other translation factors were significantly enriched in the group of genes repressed at the level of transcription, as well as in the group of genes repressed at the level of translation, indicating that their expression is strongly inhibited at various levels (Table S4, adjusted *p*-value < 10–^3^). In contrast, stress response genes were significantly enriched in the group of genes up-regulated at the level of translation; these genes were three times more likely to be in this group than expected by chance (adjusted *p*-value < 10^− 3^).

In addition to the DGE analysis, we identified genes showing significant changes in translational efficiency (TE) with the software RiboDiff [62]. Cases with increased TE were over-represented among translationally up-regulated genes, as expected, but there were also many cases that corresponded to decreased RNA-Seq signal with no significant differences at the level of Ribo-Seq (Table S5 and Figure S6). These cases are not expected to result in higher protein synthesis. We concluded that DGE provides more resolution on the possible mechanisms altering gene expression during stress.

In general, changes in ribosome density at the 5’UTR versus the CDS, in stress versus normal conditions, showed a positive correlation (Fig. 3e) and the same was true for changes in ribosome density at the uORFs versus the CDS (Figures S7 and S8). In contrast, the class of mRNAs up-regulated at the level of translation showed no such correlation, or a negative correlation in the case of *Spom.N-*, which also observed at the level of bona fide highly translated uORFs (Fig. 3f and Figure S9). In other words, in this class of genes an increase in CDS ribosome density during stress often corresponded to a decrease in uORF ribosome density, which would be consistent with uORF-mediated translational regulation. As a control we performed randomly subsampling of the same number of genes from the complete gene pool, observing that the likelihood of expecting this result by chance was very low (Figure S10, probability by chance < 10^− 3^). In contrast, other regulatory classes showed a positive correlation between changes in translation levels at the 5’UTR and CDS or no consistent trend across experiments (Figure S11). Taken together, the results support a role of uORFs in the rapid activation of stress-response genes.

### Examples of putative uORF-mediated translation regulation

**Fig. 4:**
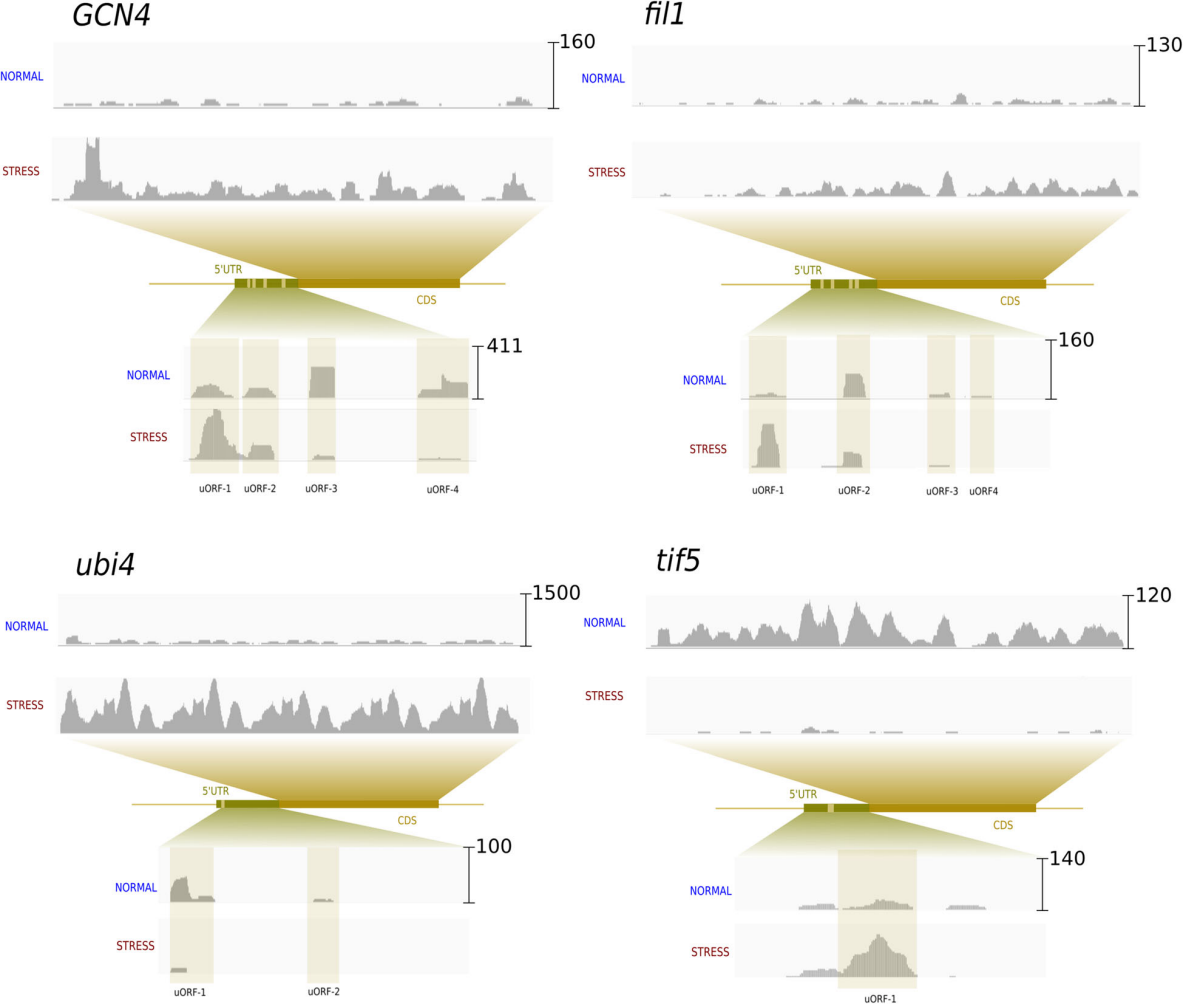
Examples of uORF-mediated translational regulation. GCN4: well-studied case in which high translation of uORFs that are proximal to the CDS has an inhibitory effect on the translation of the main protein in normal conditions. Decreased rate of translation of these uORF during stress allows the protein to be expressed. The plot shows a profile of Ribo-Seq reads in normal and stress conditions, Scer. Oxi dataset. FC CDS stress vs normal RNA-Seq: 0.84; FC CDS stress vs normal Ribo-Seq: 3.51. fi1: functional analog to GCN4 in *S. pombe*. The Fil1 mRNA shows changes in ribosome density in several uORFs, suggesting that it could also be regulated by uORFs. Data shown is from the *Spom.N-* experiment. FC CDS stress vs normal RNA-Seq: 1.01; FC CDS stress vs normal Ribo-Seq: 3.02. ubi4: the *S. cerevisiae* polyubiquitin gene Ubi4 has been involved in the response to different types of stress. Here we could observe that the translation of Ubi4 was strongly activated during nitrogen depletion in *S. pombe* and that this was accompanied by a strong decrease in the translation of two uORFs in the 5’UTR (decrease in the number of Ribo-Seq mapped reads 10–20 fold). FC CDS stress vs normal RNA-Seq: 2.07; FC CDS stress vs normal Ribo-Seq: 5.64. tif5: the translation initiation factor 5 is required for the formation of the functional 80S initiation complex and was strongly inhibited in nitrogen starvation conditions in *S. pombe*. An uORF in the 5’UTR showed much higher translation levels in stress compared to normal conditions, representing a possible example in which uORF translation contributes to specific repression of certain mRNAs during stress. FC CDS stress vs normal RNA-Seq: 0.37; FC CDS stress vs normal Ribo-Seq: 0.06

One well-studied example of a gene whose translation is known to be up-regulated by uORFs upon stress is Gcn4 [24]. Inspection of the mapped Ribo-Seq reads on the uORFs of this gene confirmed that, whereas the first two uORFs are translated at higher levels in stress, the opposite happens with the other two uORFs, permitting higher levels of translation of the CDS (Fig. 4). Below we describe other examples in which the changes in uORF ribosome occupancy also suggest uORF mediated translation regulation.

Recently, a gene that functions analogous to *Gcn4*, called *Fil1*, was discovered in *S. pombe* [17]. Our DGE analysis identified *Fil1* as a translationally up-regulated gene during starvation in *S. pombe*. Similar to *Gcn4, Fil1* showed decreased levels of translation of the most downstream uORFs during stress, suggesting that it might be regulated in a similar fashion as *Gcn4*. In both cases the first uORF showed a higher density of ribosomes during stress than in normal conditions (Fig. 4), which could potentially alter the balance at the downstream uORFs.

Another gene that showed an inverse correlation between uORF and CDS translation levels was *Ubi4* in *S. pombe.* The polyubiquitin gene *Ubi4* encodes a precursor protein that is rapidly cleaved into ubiquitin monomers after its synthesis. The gene is essential for resistance to different kinds of stress, including high temperatures, starvation and oxidative stress [18, 61]. Consistently, we found that *Ubi4* was activated upon nitrogen depletion in *S. pombe.* We observed a moderate increase in mRNA levels during stress (2-fold) and a stronger increase in translation levels (5.6-fold). This was accompanied by a decrease in the translation levels of two uORFs in the 5’UTR, suggesting that changes in the translation of these uORF might play a role in the rapid activation of *Ubi4* synthesis in stress conditions.

The induction of stress has been associated with a down-regulation of the expression of ribosomal proteins and other components of the translational machinery [19, 52] and here we observed that proteins involved in translation were over-represented among translationally down-regulated genes (Table S4). One of the strongest down-regulated genes in *S. pombe* starvation conditions was translation initiation factor 5 (*Tif5*), which is required for the formation of the functional 80S initiation complex [36, 37]. We found that the amount of ribosomes on *Tif5* coding sequence was 16 times lower during stress than during normal conditions, whereas the comparable decrease on mRNA abundance was only 2.7 fold. We identified an uORF that was translated at much higher levels during stress than in normal conditions and which could be at least in part responsible for the strong inhibition of TIF5 synthesis.

## Discussion

Recent studies based on ribosome profiling have provided strong evidence that a large number of upstream ORFs (uORFs) are translated [28, 32, 59]. The analysis of variants in thousands of individuals indicate that uORFs are under strong negative selection [55] but the functions of the majority of them are not yet well-understood. On one hand, several uORFs could have a repressive role in the translation of the downstream CDS. This has been shown in a number of well-documented cases in which inactivation of the uORF has resulted in increased CDS translation [10, 24, 30]. More generally, genome-wide studies have found that uORFs are depleted from mRNAs with respect to the random expectation and that genes containing uORFs in their 5’UTR tend to be translated less efficiently than genes that do not contain uORFs [10, 12]. On the other hand, uORFs could also encode functional proteins. This has been recently shown to be the case for several human uORFs using functional assays [11].

Here we decided to investigate the impact of uORFs in regulating changes in translation during stress using ribosome profiling data from different experiments performed in *S. cerevisiae* and *S. pombe*. By comparing data from different studies we expected to be able to increase the robustness of our conclusions. One intriguing observation made in previous studies was that ribosome density appeared to increase in the 5’UTR with respect to the CDS during stress, suggesting higher translation of uORFs [21, 27, 35]. Although cycloheximide, a translation inhibitor used in ribosome profiling experiments, could alter the distribution of ribosomes in stress vs non-stress conditions [20], it was subsequently shown than less than 5% of the genes would be affected, at least in the *S. pombe* experiments [16].

We compared ribosome density between the 5’UTR and the CDS, and also used direct mappings of Ribo-Seq reads to uORFs, selecting a subset of uORFs with clear three nucleotide periodicity. We concluded that the bias may be explained by the well-described translational arrest at the initiation codon of the CDS during stress [13, 49]. The translation of uORFs seems to be less affected by stress than the translation of the CDS, potentially increasing the relative amount of small proteins encoded by uORFs.

In general, changes in the relative translational efficiency (TE) at the 5’UTR did not affect the translation of the downstream CDS, suggesting that most uORFs do not regulate downstream translation during stress. In the same line, a recent study in human heart found that there was no clear anti-correlation in the translational efficiency of uORFs and CDS when comparing samples from patients affected by dilated cardiomyopathy versus controls [23]. Ribosome data from different Drosophila embryogenesis stages also indicated a relatively small impact of uORFs in translational regulation [42].

We found that increases in ribosome density in the CDS were positively correlated with increases in ribosome density at the uORF. The exceptions to this rule were genes up-regulated at the level of translation during stress, consistent with uORF-mediated regulation. The repression of downstream translation by uORFs may be driven by the dissociation of the ribosomal subunits upon translation termination, or to ribosome stalling, an inhibitory mechanism which depends on the interaction of small molecules and/or the peptide encoded by the uORF [25, 53]. We identified several cases in which impairment of uORF translation during stress could be related to a strong increase in protein synthesis: in addition to the well-studied *Gcn4* gene in *S. cerevisiae*, this included *Fil1* and *Ubi4* in *S. pombe*. For which to our knowledge no previous evidence of uORF-dependent translation inhibition has been reported.

The transcription of many components of the translation machinery, including ribosomal proteins and translation factors, is known to be down-regulated during stress [19, 49, 52]. Here we observed that ribosomal proteins are not only repressed at the level of transcription but also at the level of translation. We found evidence that this effect may be at least partly mediated by uORFs. A striking example was *Tif5* mRNA, which contains an uORF whose ribosome density drastically increases during stress, at the same time that *Tif5* translation decreases several fold.

The number of known regulatory uORFs is increasing although the mechanisms by which they affect CDS translation vary from gene to gene and are not always well-understood. In the case of baker’s yeast *Gcn4* four different uORFs are involved in the modulation of the translation of the main product [22, 24] but the same gene in *Candida albicans* might contain a single inhibitory uORF [50]. In the case of *S. pombe Fil1*, which is analogous to *Gcn4,* we identified four different uORFs which may shift their translation levels in response to nitrogen starvation, suggesting that the mechanism of regulation may be similar to that described in *S. cerevisiae*. Other examples in yeast include the AP1-like transcription factor encoding genes *Yap1* and *Yap2*, which contain a variable number of uORFs modulating their translation [9]. These uORFs were not analyzed in the present study because the Ribo-Seq signal was too weak for further statistical analysis. A role of uORFs in the regulation of genes during arsenite treatment of human cells has also been proposed [2]. In other cases, uORFs may be required to maintain the levels of the main protein under control; for example, loss of an uORF in human tyrosine kinase mRNA leads to over-expression of the protein and oncogeneicity [54]. Large-scale analysis of mutations in cancer has identified several uORF deletions that could be associated with protein expression dysregulation and malignancy [48] and a number of genetic diseases are caused by mutations that introduce or eliminate uORFs and change the translational efficiency of the main coding sequence [3].

Whereas a large proportion of cellular mRNAs contain uORFs, it has remained unclear how many of them have a regulatory role. Our results suggest that many of them are likely to be permissive for downstream translation, perhaps because, as the first two uORFs in *Gcn4*, they allow ribosome scanning re-initiation. Additionally, some of the uORFs might encode micropeptides that are functional per se. Although uORF sequences tend to show poor phylogenetic conservation [59], several functional peptides encoded by uORFs have been recently described in humans [11]. Some of these uORF-encoded peptides interact directly with the main protein; in this regard, co-expression from the same mRNA could facilitate the neo-functionalization of peptides encoded by novel uORFs and their integration in cellular pathways related to the main protein product. If this is so, these mRNAs could be considered bicistronic transcripts, encoding two different functional protein products.

Although ribosome profiling experiments can be used to detect three nucleotide periodicity patterns in uORFs that indicate active translation, uORFs are very small compared to CDS [15], and their translation may in some cases be difficult to determine. The results of the analyses we perform with the complete set of uORFs, and the subset of uORFs for which we could verify translation using three nucleotide periodicity, did not show clear differences in any of the variables measured, which could mean that the majority of uORFs are translated, even if this translation is sometimes difficult to detect.

A possible limitation of our study is that we only analyzed AUG initiation codons, whereas other analyses have concluded that non-AUG codons may also initiate uORF translation [27]. Our choice was motivated by previous experiments that compared the translation initiation efficiency of different codons in yeast and concluded that the efficiency of non-AUG codons was very low compared to AUG codons [14, 64]. In other words, we decided to favour specificity over sensitivity. Another possible limitation was that no complete annotations for *S. cerevisiae* 5’UTR sequences existed. To compensate for this, we combined data from five different 5’UTR annotation studies, obtaining 5’UTR sequences for about half of the annotated genes. In the case of *S. pombe* the available annotation files include 5’UTR coordinates for virtually all genes, which facilitated the exhaustive identification of uORFs and the detection of bona fide translated ones.

## Conclusions

Our results suggest that, although uORFs are translated at higher levels than the CDS in stress, the majority of them might not repress the main coding sequence. The exception are uORFs in a number of key stress-response genes, which show anti-correlated translation levels with respect to the CDS. Our observations open new questions about the evolution and function of uORFs.

## Methods

### Sequencing data

We downloaded RNA-Seq and Ribo-Seq sequencing reads from three published experiments in which stress was induced to the cells in the culture. The first one, which we named *Scer.aa-*, was an amino acid depletion experiment performed in *S. cerevisiae* [27]. In this experiment the cells were transfered from a rich medium (YPD) to a minimal medium (SD) without amino acids for 20 min. Thus, the observed patterns can be due to lack of amino acids but also other differences in the media, including lack of other nutrients. The second one, *Scer. Oxi* was an oxidative stress experiment also perfomed in *S. cerevisiae* [21]. In this case 0.2 mM hydrogen peroxide (H_2_O_2_) was added to the medium for 5 or 30 min. To simplify here we only used the cells treated for 30 min, which showed a stronger increase in the proportion of reads that mapped to the 5’UTR. In the third experiment, *Spom.N-*, nitrogen was depleted from the medium [16]. According to the authors, the *S. pombe* cells were grown in Edinburgh Minimal Medium 2 (EMM2) containing 93.4 mM NH_4_Cl before moving them to the same medium without NH_4_Cl for 60 min. We obtained available RNA-Seq and Ribo-Seq (ribosome profiling) data for the three experiments, both for treated and untreated cells. The sequencing data identifiers for *Scer.aa*- and *Spom.N-* can be found in Table S6, data for *Scer. Oxi* was directly provided by the authors. We used two replicates per condition and experiment as some experiments did not have more than two replicates.

We performed RNA-Seq sequencing read quality filtering with cutadapt v1.16 [38] and used FastQC v0.11.5 (https://www.bioinformatics.babraham.ac.uk/projects/fastqc/) to assess the quality of the reads. In the case of Ribo-Seq we also removed ribosomal RNA (rRNA). For this we selected the coordinates of all rRNA features in the corresponding gene annotation files. We used gffread (https://github.com/gpertea/gffread) to create files containing the rRNA sequences and subsequently eliminated the reads that mapped to these sequences.

### mRNA read mapping and quantification

RNA-Seq and Ribo-Seq sequencing reads were mapped to the genome using Bowtie2 [33]; genome sequences were retrieved from Ensembl (version 39 for *S. pombe* and 92 for *S. cerevisiae*). We generated separate annotation files for coding sequences (CDS) and 5′ untranslated regions (5’UTR). In the case of *S. pombe* these two files were obtained using the ‘CDS’ and ‘5UTR’ labels in the annotation file to separate out the entries. In the case of *S. cerevisiae* the CDS annotation file was generated in the same manner. As virtually no information on 5’UTR coordinates is available from the standard *S. cerevisiae* annotation file we built our own 5’UTR annotation file combining data from five previously published studies [39, 41, 57, 58, 60]. This file contained a non-redundant set of 5’UTRs; when several 5’UTR annotations existed for the same transcript we took the longest one.

After read mapping we generated the corresponding tables of counts, containing the number of reads mapping to each feature in each sequencing sample. For this we used HTSeq-count [1] with parameters: “htseq-count -s <yes/no> -a 0 -t exon -i gene_id”. Additionally, for CDS we used the htseq-count parameter “-m union”, whereas for 5’UTR we used “-m intersection_strict”. The latter condition is more restrictive and was used to eliminate reads that could correspond to ribosomes located on the first bases of the CDS instead of the 5’UTR.

### Identification of uORFs and translation quantification

We identified all possible upstream ORFs (uORFs) within the 5’UTRs starting with ATG and ending with a STOP codon (available as supplementary material). We focused on canonical uORFs with an AUG start codon, which are expected to be translated more efficiently than those initiating with near-cognate codons (NCCs) [63]**.** We then used RibORF [29] to count the number of Ribo-Seq reads that mapped to the P-site in each uORF sequence. We normalized the number of Ribo-Seq reads mapped to each uORF by Million mapped reads, obtaining the counts per Million (CPM). The fold change (FC) of each uORF between conditions was calculated as the CPM in stress divided by the CPM in normal conditions, taking the average between the replicates; we then applied a logarithmic transformation to obtain the log_2_FC. In the *Scer. Oxi* dataset uORF table of counts we only used values of one of the replicates because the other one had a very low number of reads. For further analysis we selected uORFs with a minimum length of 9 amino acids and at least 10 mapped reads considering all samples together. We identified 44 such uORFs in *Scer.aa-*, 196 in *Scer. Oxi* and 1500 in *Spom.N-*.

We also used the RibORF pipeline to select a subset of uORFs containing strong signatures of selection on the basis of three nucleotide periodicity and homogeneity of the reads along the uORF. In the RibORF output reads in frame 1 (f1) correspond to the in-frame reading sequence; an excess of such reads with respect to reads in frames 2 and 3 (f2 and f3) indicates a pattern of three nucleotide periodicity, consistent with translation. We selected uORFs with a RibORF score > 0.7, as a set of bona fide translated uORFs. The RibORF score cut-off was chosen on the basis of previous studies showing that it was associated with a false discovery rate lower than 0.05 [29, 46].

### Ratio between the number of reads in the 5’UTR/uORF and the CDS

We calculated the average value of the two replicates in the tables of counts of CDS and 5’UTR, both for Ribo-Seq and RNA-Seq data. We removed genes if both average values (normal and stress) were below 10 reads. Subsequently we calculated the ratio between 5’UTR and CDS average values, in stress and normal conditions. In the case of uORFs we used RibORF to map the reads to the P-site and selected uORFs with at least 10 mapped Ribo-Seq reads taking all samples together. Once we had this information we separated out 5’UTRs that contained putatively translated uORFs to those that did not.

### Measuring changes in the relative number of reads in stress vs normal conditions

In order to compare the relative changes in ribosome density in stress versus normal conditions for each gene we normalized the counts to counts per Million (CPM), by dividing by one Million mapped reads. The fold change (FC) of each gene between conditions was then calculated as the CPM in stress divided by the CPM in normal conditions, taking the average between the replicates. We then applied a logarithmic transformation to obtain the log_2_FC, in which positive values correspond to higher expression of that gene in stress than in normal conditions and negative values the other way round, relative to other genes.

### Translational efficiency

We calculated the translational efficiency (TE) of each sequence by dividing the Ribo-Seq CPM values to the RNA-Seq CPM values. The TE fold change (FC) was then calculated as TE in stress divided by TE in normal conditions. We used RiboDiff [62]⁠ to identify genes that showed significant changes in TE between stress and normal conditions (adjusted *p*-value 0.05).

### Differential gene expression analysis

The identification of genes that are significantly up-regulated or down-regulated using RNA-Seq and Ribo-Seq data can be used to differentiate between genes that are likely to be regulated at the level of transcription (both RNA-Seq and Ribo-Seq show the same tendency) from those that are regulated primarily at the level of translation (significantly up-regulated or down-regulated by Ribo-Seq but not RNA-Seq data), or that undergo post-transcriptional buffering of gene expression (only significant by RNA-Seq) [26]. In order to perform differential gene expressoin (DGE) analysis for each experiment we subsampled the CDS table of counts so as to have approximately the same number of mapped reads in each of the samples. This step ensured we would have similar statistical power when using the RNA-Seq or Ribo-Seq data. We then normalized the data using the Trimmed Mean of M-values (TMM) algorithm from the R/Bioconductor package edgeR [43]. Subsequently, we used the *limma-voom* method to determine which genes showed significant changes in abundance in stress conditions [34], separately for RNA-Seq and Ribo-Seq data. Significantly up-regulated or down-regulated genes were those with adjusted p-value lower than 0.05 and log_2_FC greater than one standard deviation (SD) of the log_2_FC distribution for the corresponding data. The SD values were as follows: *Scer.aa-* Ribo-Seq: 0.99 and RNA-Seq: 0.87; *Scer. Oxi* Ribo-Seq: 1.53 and RNA-Seq: 1.47; *Spom.N-* Ribo-Seq: 1.38 and RNA-Seq: 1.01.

### Gene ontology term enrichment

We calculated the enrichment in Gene Ontology (GO) terms of the Biological Process category in different subsets of *S. pombe* genes that showed specific regulatory patterns. We selected representative terms that were significantly over-represented in the set of interest with FDR < 0.01. For this we used the AnGeLi webserver application from the Bähler Lab at University College London (http://bahlerweb.cs.ucl.ac.uk/cgi-bin/GLA/GLA_input).

## Supplementary Information

**Additional file 1: Table S1.** Number of genes with higher number of reads in the 5’UTR vs CDS in stress than in normal conditions (S/*N* > 0) and the other way round (S/*N* < 0). **Table S2.** Functional term analysis for genes with uORFs. **Table S3.** Defining the type of gene regulation using DGE data from RNA-Seq and Ribo-Seq. **Table S4.** Gene Ontology term enrichment for genes regulated during stress. **Table S5.** Analysis of genes showing significant changes in translational efficiency (TE) between stress and normal conditions. **Table S6.** Sequence datasets used in the study. **Figure S1.** Log_10_ ratio of 5’UTR to CDS RNA-Seq reads in stress versus normal conditions in the three experiments. **Figure S2.** Log_10_ ratio of translated uORF to CDS Ribo-Seq reads in stress versus normal conditions in the three experiments. **Figure S3.** Three nucleotide periodicity of uORF mapped Ribo-Seq reads. **Figure S4.** Changes in translational efficiency (TE) at the 5’UTR versus the CDS. **Figure S5.** Pairwise Spearman correlation values in the number of mapped reads per gene. **Figure S6.** Enrichment in mRNAs with increased or decreased TE in different DGE classes. **Figure S7.** Ribosome density at the uORFs and downstream CDS is positively correlated. **Figure S8.** Ribosome density at the uORFs and downstream CDS is positively correlated. **Figure S9.** Comparison of changes in Ribo-Seq mapped reads in CDS and translated uORFs, for genes upregulated at the level of translation. **Figure S10.** Random subsampling of genes with the same number of data-points as datasets in Fig. 2. **Figure S11.** Comparison of changes in Ribo-Seq mapped reads for different gene sets.

## Data Availability

Additional file 1 contains supplementary tables and figures mentioned in the text. The datasets supporting the conclusions of this article are available in the Figshare repository [10.6084/m9.figshare.12320597.v2].
