# Supplementary material for: Impact of uORFs in mediating regulation of translation in stress conditions
Source: BMC Mol Cell Biol. 2021 May 16;22:29. doi: 10.1186/s12860-021-00363-9 (PMC8126119; doi:10.1186/s12860-021-00363-9)
Supplement: Supplementary file 1 — Additional file 1: Table S1. Number of genes with higher number of reads in the 5’UTR vs CDS in stress than in normal conditions (S/N > 0) and the other way round (S/N < 0). Table S2. Functional term analysis for genes with uORFs. Table S3. Defining the type of gene regulation using DGE data from RNA-Seq and Ribo-Seq. Table S4. Gene Ontology term enrichment for genes regulated during stress. Table S5. Analysis of genes showing significant changes in translational efficiency (TE) between stress and normal conditions. Table S6. Sequence datasets used in the study. Figure S1. Log10 ratio of 5’UTR to CDS RNA-Seq reads in stress versus normal conditions in the three experiments. Figure S2. Log10 ratio of translated uORF to CDS Ribo-Seq reads in stress versus normal conditions in the three experiments. Figure S3. Three nucleotide periodicity of uORF mapped Ribo-Seq reads. Figure S4. Changes in translational efficiency (TE) at the 5’UTR versus the CDS. Figure S5. Pairwise Spearman correlation values in the number of mapped reads per gene. Figure S6. Enrichment in mRNAs with increased or decreased TE in different DGE classes. Figure S7. Ribosome density at the uORFs and downstream CDS is positively correlated. Figure S8. Ribosome density at the uORFs and downstream CDS is positively correlated. Figure S9. Comparison of changes in Ribo-Seq mapped reads in CDS and translated uORFs, for genes upregulated at the level of translation. Figure S10. Random subsampling of genes with the same number of data-points as datasets in Fig. 2. Figure S11. Comparison of changes in Ribo-Seq mapped reads for different gene sets. [file 12860_2021_363_MOESM1_ESM.pdf]

Moro et al. Impact of uORFs in mediating regulation of translation in stress conditions. Additional file 1 containing supplementary Tables and Figures.

## Table S1

| | Spom.N- | | | Scer.Oxi | | | Scer.aa- | | |
|---|---|---|---|---|---|---|---|---|---|
| | **S/N > 0** | **S/N < 0** | **Total** | **S/N > 0** | **S/N < 0** | **Total** | **S/N > 0** | **S/N < 0** | **Total** |
| **1. All – RiboSeq** | 3,289 (86,2%) | 526 (13,8%) | 3,816 | 1,999 (91.4%) | 189 (8.6%) | 2,188 | 1,678 (92.1%) | 144 (7.9%) | 1,822 |
| **2. All - RNASeq** | 1,765 (48.9%) | 1,843 (51.1%) | 3,608 | 1,423 (59.7%) | 959 (40.3%) | 2,382 | 925 (41.1%) | 1,324 (58.9%) | 2,249 |
| **3. 5'UTR no uORFs - RiboSeq** | 2,450 (86%) | 398 (14%) | 2,849 | 1,880 (91.6%) | 173 (8.4%) | 2,053 | 1,649 (92.2%) | 139 (7.8%) | 1,788 |
| **4. 5'UTR with uORFs - RiboSeq** | 839 (86.8%) | 128 (13.2%) | 967 | 119 (88.1%) | 16 (11.9%) | 135 | 29 (85.3%) | 5 (14.7%) | 34 |
| **5. 5'UTR with *bona fide* translated uORFs - RiboSeq** | 286 (89.9%) | 32 (10.1%) | 318 | NA | NA | NA | NA | NA | NA |

**Table S1. Number of genes with higher number of reads in the 5'UTR vs CDS in stress than in normal conditions (S/N>0) and the other way round (S/N < 0).** S/N= $\log2((5'UTR/CDS)_{stress}/(5UTR/CDS))_{normal}$, where 5'UTR is the number of mapped Ribo-Seq reads in the 5'UTR region and CDS the number of Ribo-Seq reads in the CDS region. Also shown is the total number of genes analyzed (including S/N=0). No uORFs -RiboSeq refers to mRNAs in which the 5'UTR contains no translatable uORFs. Note that we only considered mRNAs with a minimum of 10 mapped reads, taking the average between the replicates, in at least one of the conditions. The proportions in 1, 3, 4 and 5 within the same experiment showed no significant differences according to a Fisher test (p> 0.05), denoting that the presence of uORFs does not have a significant impact in the number of cases in which the 5'UTR/CDS ratio is higher in stress than in normal conditions. *Bona fide* translated uORFs were predicted on the basis of high three nucleotide periodicity and homogeneity of the Ribo-Seq reads along the ORF, using the RibORF program (score > 0.7). Only data for *Spom.N-* dataset is shown because the other datasets did not comprise a sufficient number of cases to ensure robust statistical analysis (NA: non-applicable). The data is related to Figure 1 in the main manuscript file and Figures S1 and S2.

| Dataset | Cluster | Observed | Expected | FDR | status |
|---|---|---|---|---|---|
| 5'UTR contains uORFs | | | | | |
| | translation (GO Biological process) | 4.02 % (40/996) | 7.81% (401/5136) | 4.2e-05 | Under-representation |
| | Highly expressed genes (Expression) | 3.61 % (36/996) | 8.90 % (457/5136) | 9.65e-10 | Under-representation |
| | core environmental stress response repressed (Expression) | 2.61 % (26/996) | 8.55 % (439/5136) | 1.31e-13 | Under-representation |
| | oxidative stress cluster 3 (Expression) | 9.04% (90/996) | 5.76% (296/5136) | 0.00053 | Over-representation |

**Table S2. Functional term analysis for genes with uORFs.** We investigated the over-representation or under-representation of Gene Ontology terms and Expression Clusters in the set of *S. pombe* genes with uORFs. We focused on potentially translated uORFs covered by 10 or more Ribo-Seq reads taking all samples together. The enrichment was calculated using a Fisher test and false discovery rate (FDR) correction. We are showing the most representative clusters with FDR < 0.01 that had at least 5% frequency in the general gene set. The analysis was performed with AnGeLi (Analysis of Gene Lists) web server application from the Bähler Lab at University College London (http://bahlerweb.cs.ucl.ac.uk/cgi-bin/GLA/GLA_input).

| Gene set | Scer.aa- | Scer.Oxi | Spom.N- | class |
|---|---|---|---|---|
| RNA-UP/RIBO-UP | 161 | 310 | 295 | Transcriptionally upregulated |
| RNA-DOWN/RIBO-DOWN | 294 | 585 | 228 | Transcriptionally downregulated |
| RIBO-UP/RNA ns | 114 | 154 | 194 | Translationally upregulated |
| RIBO-DOWN/RNA ns | 83 | 152 | 363 | Translationally downregulated |
| RNA-UP/RIBO ns | 159 | 127 | 49 | Postranscriptional buffering |
| RNA-DOWN/RIBO ns | 129 | 144 | 38 | Postranscriptional buffering |
| RNA-UP/Ribo-DOWN | 0 | 0 | 0 | - |
| RNA-DOWN/Ribo-UP | 1 | 1 | 0 | - |
| RNA & RIBO ns | 1939 | 3713 | 3383 | - |
| TOTAL | 2880 | 5186 | 4550 | |

**Table S3. Defining the type of gene regulation using DGE data from RNA-Seq and Ribo-Seq.** The number of genes in each type and experiment is shown. ns: non-significant in the DGE analysis. RNA UP – RIBO UP: transcriptional upregulation during stress; RNA DOWN – RIBO DOWN: transcriptional downregulation during stress; RIBO UP- RNA ns: genes up-regulated at the level of translation during stress (translational UP stress); RIBO DOWN – RNA ns: genes down-regulated at the level of translation during stress (translational DOWN stress); RNA DOWN – RIBO ns: post-transcriptional buffering; RNA UP – RIBO ns: post-transcriptional buffering.

| Dataset | GO Biological process | Observed | Expected | FDR |
|---|---|---|---|---|
| Translational UP stress | | | | |
| | Core Environmental Stress Response induced | 34.54 % (67/194) | 10.44 % (536/5136) | 1.64984e-16 |
| | Oxidative Stress Cluster 4 | 26.8 % (52/194) | 8 % (411/5136) | 3.37152e-12 |
| Translational DOWN stress | | | | |
| | cytoplasmic translation | 28.65 % (104/363) | 4.77 % (245/5136) | 1.72897e-55 |
| | rRNA metabolic process | 16.25 % (59/363) | 4.23 % (217/5136) | 2.93907e-18 |
| | biosynthetic process | 53.44 % (194/363) | 32.67 % (1678/5136) | 9.46996e-15 |
| Transcriptional UP stress | | | | |
| | amino acid transport | 4.41 % (13/295) | 0.76 % (39/5136) | 3.87e-05 |
| Transcriptional DOWN stress | | | | |
| | cytoplasmic translation | 31.28 % (71/227) | 4.77 % (245/5136) | 6.8e-39 |
| Postranscriptional buffering UP stress | | | | |
| | Core Environmental Stress Response induced | 55.1 % (27/49) | 10.44 % (536/5136) | 8.74e-11 |
| | Oxidative Stress Cluster 4 | 42.86 % (21/49) | 8 % (411/5136) | 1.26e-07 |
| | Reproduction module | 26.53 % (13/49) | 5.74 % (295/5136) | 0.0071 |
| Postranscriptional buffering DOWN stress | | | | |
| | ribosome biogenesis | 39.47% (15/38) | 6.93% (356/5136) | 2.54251e-05 |
| | rRNA metabolic process | 26.32% (10/38) | 4.23% (217/5136) | 0.00143553 |

**Table S4. Gene Ontology term enrichment for genes regulated during stress.** Analysis of genes classified in different regulatory modes according to DGE analysis of the *Spom.N-* dataset. The enrichment was calculated using a Fisher test and false discovery rate (FDR) correction. Terms with a FDR < 0.01 were retrieved; we filtered out highly redundant terms and took the largest one as the representative. The analysis was performed with AnGeLi (Analysis of Gene Lists) web server application from the Bähler Lab at University College London (http://bahlerweb.cs.ucl.ac.uk/cgi-bin/GLA/GLA_input).

**Scer.aa-**

| | RIBO UP - RNA ns | RIBO DOWN- RNA ns | RNA DOWN – RIBO ns | RNA UP – RIBO ns | RIBO UP - RNA UP | RNA DOWN- RIBO DOWN | RNA UP - RIBO DOWN | RIBO UP - RNA DOWN | ns | Total |
|---|---|---|---|---|---|---|---|---|---|---|
| Increased TE | **13** | 0 | 7 | 0 | 11 | 1 | 0 | 1 | 2 | **35** |
| Decreased TE | 0 | **9** | 0 | 18 | 4 | 9 | 0 | 0 | 6 | **46** |
| All genes DGE analysis | 114 | 83 | 129 | 159 | 161 | 294 | 0 | 1 | 1939 | 2880 |

**Scer.Oxi**

| | RIBO UP - RNA ns | RIBO DOWN- RNA ns | RNA DOWN – RIBO ns | RNA UP – RIBO ns | RIBO UP - RNA UP | RNA DOWN – RIBO DOWN | RNA UP - RIBO DOWN | RIBO UP - RNA DOWN | ns | Total |
|---|---|---|---|---|---|---|---|---|---|---|
| Increased TE | **76** | 0 | 86 | 0 | 55 | 89 | 0 | 1 | 509 | **816** |
| Decreased TE | 0 | **115** | 0 | 84 | 33 | 84 | 0 | 0 | 615 | **931** |
| All genes DGE analysis | 154 | 152 | 144 | 127 | 310 | 585 | 0 | 1 | 3713 | 5186 |

**Spom.N-**

| | RIBO UP - RNA ns | RIBO DOWN- RNA ns | RNA DOWN – RIBO ns | RNA UP – RIBO ns | RIBO UP - RNA UP | RNA DOWN – RIBO DOWN | RNA UP - RIBO DOWN | RIBO UP - RNA DOWN | ns | Total |
|---|---|---|---|---|---|---|---|---|---|---|
| Increased TE | **33** | 0 | 0 | 0 | 30 | 0 | 0 | 0 | 5 | **68** |
| Decreased TE | 0 | **80** | 0 | 3 | 0 | 45 | 0 | 0 | 34 | **162** |
| All genes DGE analysis | 194 | 363 | 38 | 49 | 295 | 228 | 0 | 0 | 3383 | 4550 |

**Table S5. Analysis of genes showing significant changes in translational efficiency (TE) between stress and normal conditions.** TE: translation efficiency. Increased or decreased TE refers to genes that show significant relative changes in TE in stress versus normal (Ribodiff, FDR < 0.05). Number of genes analyzed: *Scer.aa-* 2,880, *Scer.Oxi* 5,186, *Spom.N-* 4,550. Differential gene expression (DGE) analysis defined different regulatory gene types (see main manuscript file for details on the methods). RIBO UP- RNA ns: genes up-regulated at the level of translation during stress (translational UP stress); RIBO DOWN – RNA ns: genes down-regulated at the level of translation during stress (translational DOWN stress); RNA DOWN – RIBO ns: post-transcriptional buffering downregulation during stress; RNA UP – RIBO ns: post-transcriptional buffering upregulation during stress; RNA UP – RIBO UP: transcriptional upregulation during stress; RNA DOWN – RIBO DOWN: transcriptional downregulation during stress. ns: non-significant in the DGE analysis.

| Dataset | Sequence database | Ribo-Seq N1 | Ribo-Seq N2 | Ribo-Seq S1 | Ribo-Seq S2 | RNA-Seq N1 | RNA-Seq N2 | RNA-Seq S1 | RNA-Seq S2 | Reference |
|---|---|---|---|---|---|---|---|---|---|---|
| Scer.aa- | GEO | SRR014374<br>SRR014375<br>SRR014376 | SRR014377<br>SRR014378<br>SRR014379<br>SRR014380<br>SRR014381 | SRR014368<br>SRR014369 | SRR014370<br>SRR014371<br>SRR014372<br>SRR014373 | SRR014385 | SRR014386<br>SRR014387<br>SRR028774 | SRR014382 | SRR014383<br>SRR014384 | Ingolia et al., 2009 |
| Spom.N- | ArrayExpress | ERR1994961 | ERR1994962 | ERR1994969 | ERR1994970 | ERR1994959 | ERR1994960 | ERR1994967 | ERR1994968 | Duncan and Mata, 2018 |

**Table S6. Sequence datasets used in the study.** The source of the raw sequencing data is provided. N1: normal, replicate 1; N2: normal, replicate 2; S1: stress, replicate 1; S2: stress, replicate 2. In the case of *Scer.Oxi* the sequencing data was obtained from the authors.

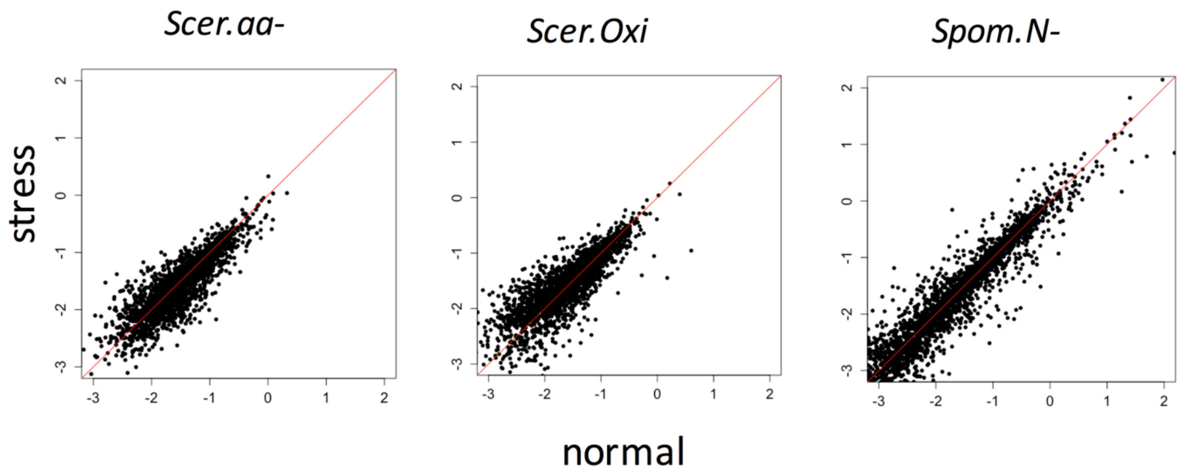


**Figure S1. Log$_{10}$ ratio of 5'UTR to CDS RNA-Seq reads in stress versus normal conditions in the three experiments.** In each sample the average was taken for replicates of the same experiment. Y axis: ratio of the 5'UTR and the downstream CDS number of counts in stress; X axis: ratio of the 5'UTR and the downstream CDS number of counts in normal conditions.

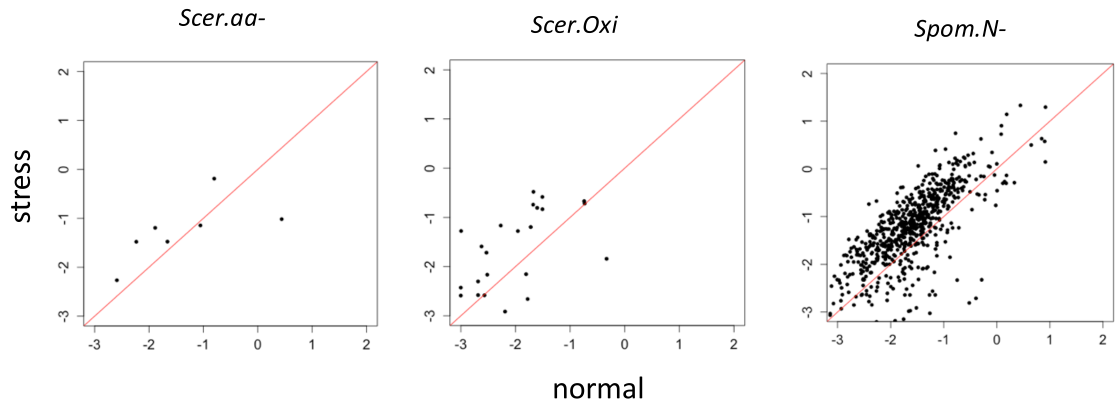


**Figure S2. Log$_{10}$ ratio of translated uORF to CDS Ribo-Seq reads in stress versus normal conditions in the three experiments.** Translated uORFs were predicted from the Ribo-Seq information in all samples taken together and using the software RibORF. uORFs with a RibORF score > 0.7 were selected. In each sample the average was taken for replicates of the same experiment. Y axis: ratio of the uORF and the downstream CDS number of counts in stress; X axis: ratio of the uORF and the downstream CDS number of counts in normal conditions. When several uORFs existed on the same mRNA we considered all of them separately. Number of datapoints: *Scer.aa-* 7; *Scer.Oxi* 29; *Spom.N-* 664.

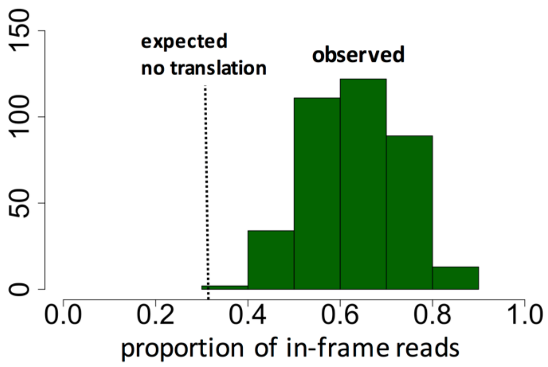


**Figure S3. Three nucleotide periodicity of uORF mapped Ribo-Seq reads.** Proportion of in-frame Ribo-Seq reads when we selected uORFs from the *Spom.N-* dataset with more than 50 mapped Ribo-Seq reads and a RibORF score higher than 0.7. Number of uORFs: 371; median observed: 0.636; expected no translation 0.333.

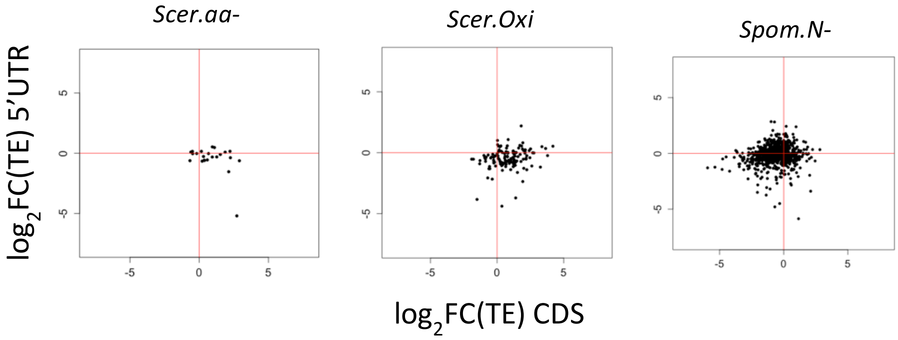


**Figure S4. Changes in translational efficiency (TE) at the 5'UTR versus the CDS.** Data is for 5'UTRs containing putatively translated uORFs. These uORFs had at least 10 mapped Ribo-Seq reads considering all samples together. We discarded genes with less than 10 mapped reads in both stress and normal condtions, taking the average between replicates, for Ribo-Seq and/or RNA-Seq experiments. Number of datapoints: *Scer.aa-* 23, *Scer.Oxi* 130, *Spom.N-* 797.

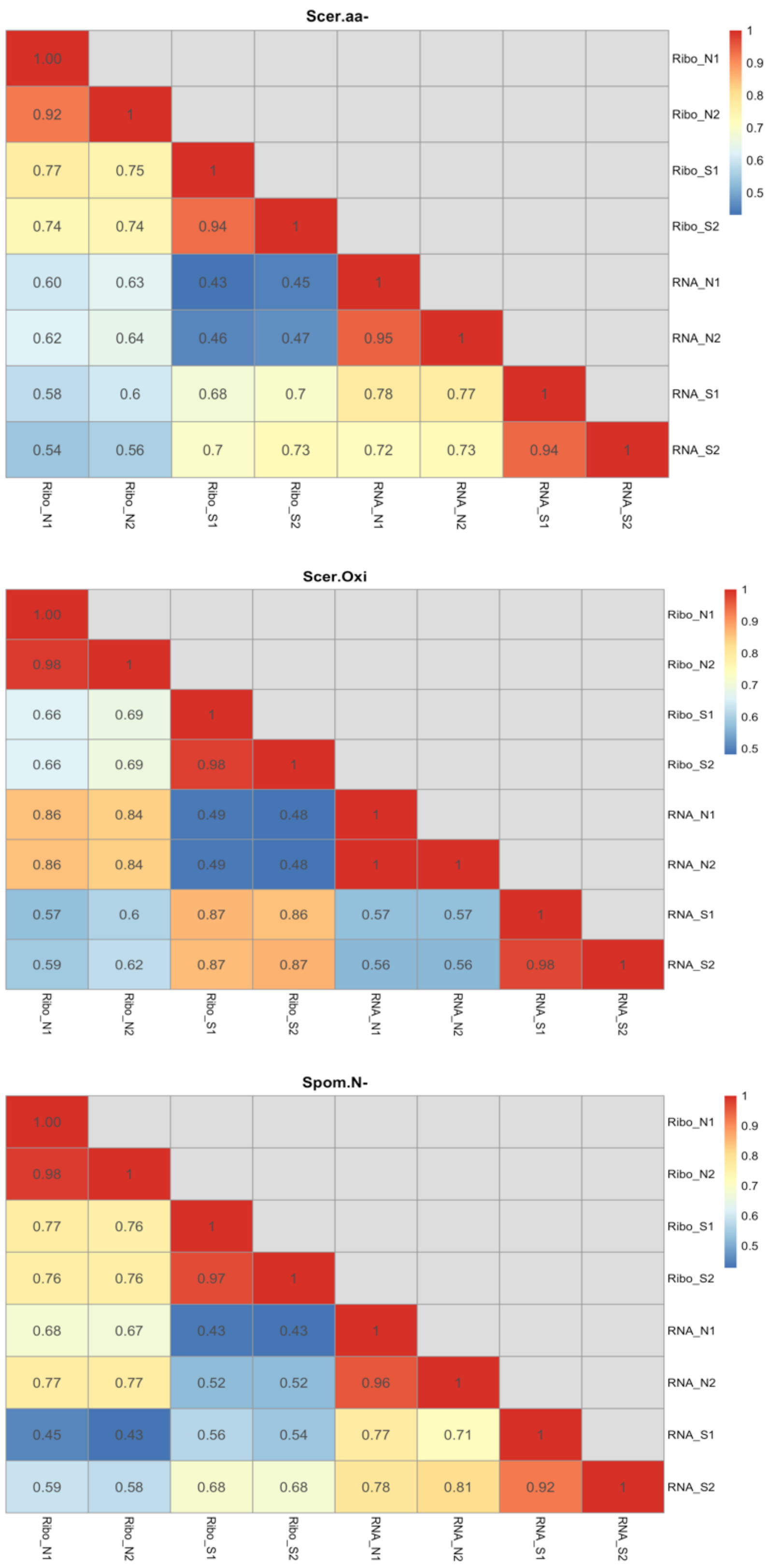


**Figure S5. Pairwise Spearman correlation values in the number of mapped reads per gene.** RNA: RNA-Seq; Ribo:Ribo-Seq. The values refer to the reads mapped to the CDS. Subsampled tables of counts were used (DGE analysis). N: normal condition; S: stress condition.

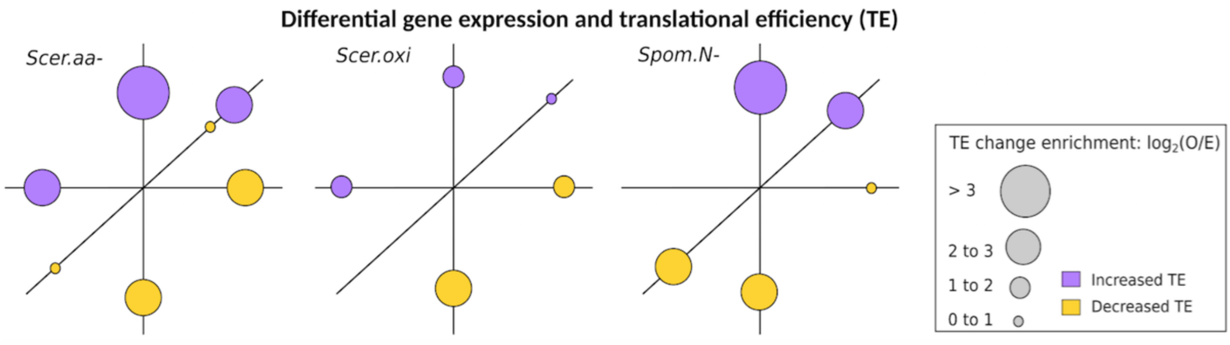


**Figure S6. Enrichment in mRNAs with increased or decreased TE in different DGE classes.** We first defined genes with significantly increased TE or significantly decreased TE using RiboDiff (FDR<0.05). We then calculated the enrichment in these genes in the previously defined DGE classes (see Figure 2 in the main manuscript file). The enrichment was calculated as the $log_2$ of the observed versus expected frequencies (see Table S4).

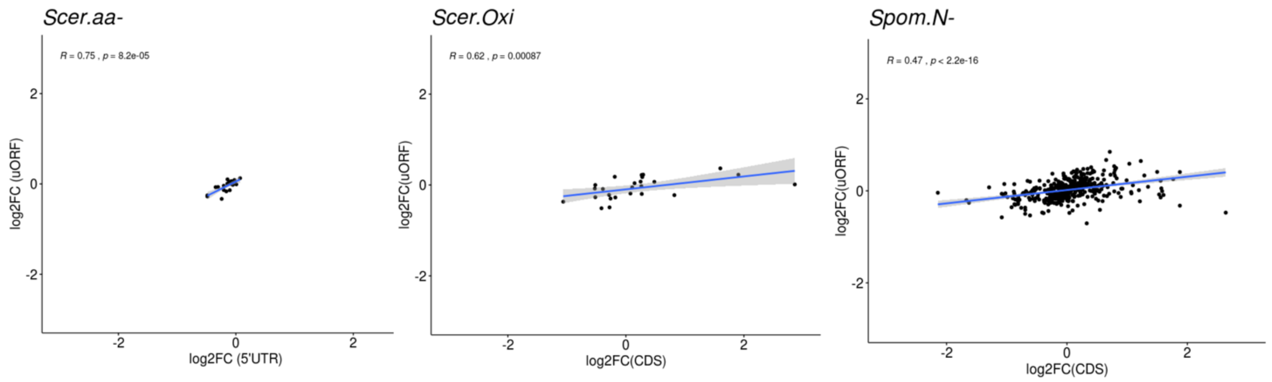


**Figure S7. Ribosome density at the uORFs and downstream CDS is positively correlated.** All mRNAs with uORFs were included. log2FC is the log2 of the ratio between the normalized Ribo-Seq reads at the 5'UTR and the CDS in stress conditions divided by the same ratio in normal conditions. Spearman correlation values are provided.

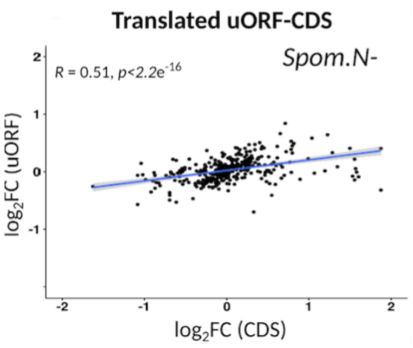


**Figure S8. Ribosome density at the uORFs and downstream CDS is positively correlated.** Data is for mRNAs containing *bona fide* highly translated uORFs in the Spom.N- dataset (number of mapped Ribo-Seq reads > 50, RibORF score > 0.7). log2FC is the log2 of the ratio between the normalized Ribo-Seq reads at the 5'UTR and the CDS in stress conditions divided by the same ratio in normal conditions. Spearman correlation is provided.

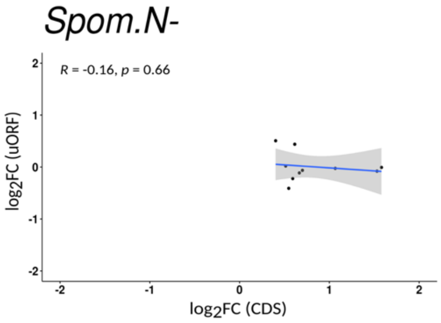


**Figure S9. Comparison of changes in Ribo-Seq mapped reads in CDS and translated uORFs, for genes up-regulated at the level of translation.** There is a lack of positive correlation in ribosome density changes between stress and normal conditions for CDS and *bona fide* highly translated uORFs. Data is for uORFs in the Spom.N- dataset with number of mapped Ribo-Seq reads > 50 and RibORF score > 0.7.

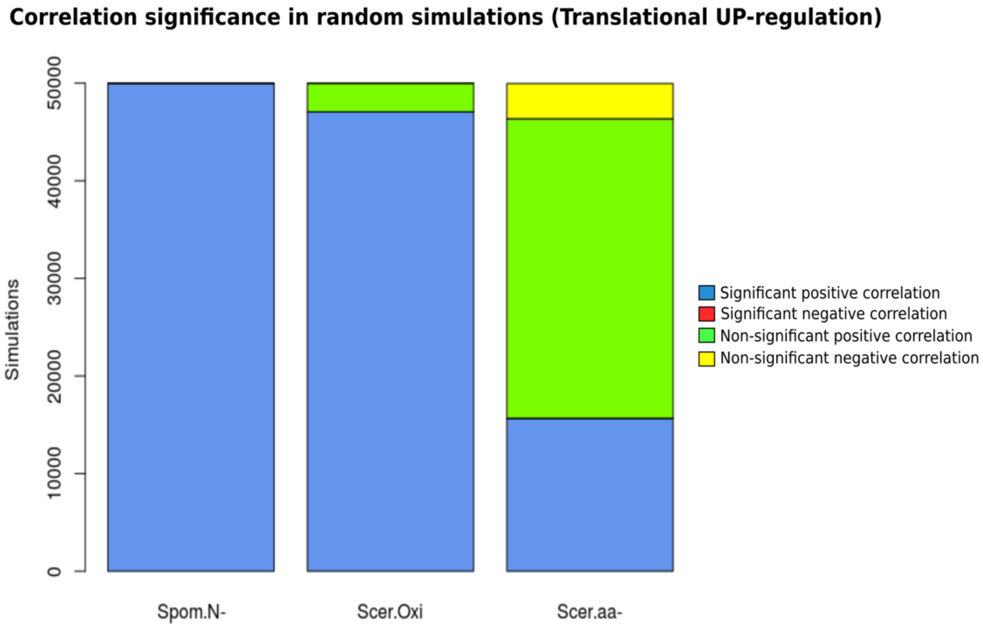


**Figure S10. Random subsampling of genes with the same number of data-points as datasets in Figure 2.** We performed 50,000 random simulations taking the same number of genes as in Figure 4A and measuring the correlation of the logFC between the number of mapped reads in 5'UTR and CDS. In most cases we obtained a significant positive correlation (in blue, significant at p-value < 0.05). In the experiment with the largest number of 5'UTR sequences, Spom.N-, we did not observe any case with significant negative correlation, as observed with real genes, indicating that the likelihood of observing our result by chance is less than $10^{-3}$. In the experiment with the lowest number of 5'UTR sequences with mapped reads, Scer.aa-, we obtained many non-significant results due to the small samples size and the results were not conclusive.

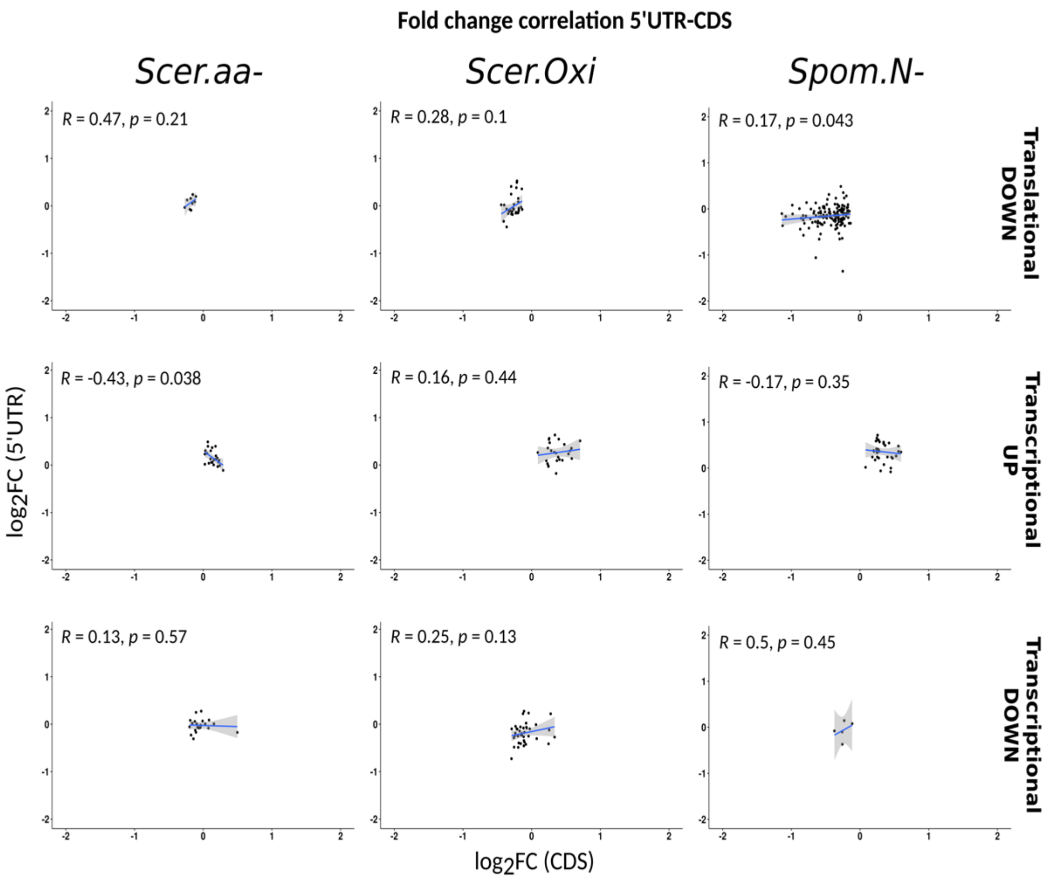


**Figure S11. Comparison of changes in Ribo-Seq mapped reads for different gene sets.** The sets of genes 'translational DOWN' and 'transcriptional DOWN' show a good agreement with the general trend of positive correlation in the changes in ribosome density between the CDS and the 5'UTR. The set of genes 'Transcriptional UP' does not show a consistent pattern across experiments.
